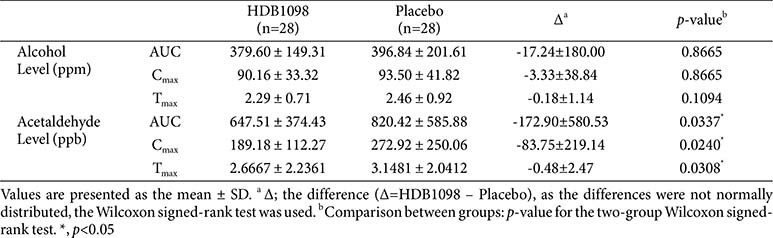# Effects of Paraprobiotic *Limosilactobacillus fermentum* HDB1098 on Hangover Improvement in Humans: A Randomized Double-Blind Placebo-Controlled Crossover Clinical Trial

**DOI:** 10.4014/jmb.2508.08003

**Published:** 2025-10-27

**Authors:** Yoo Kyung Kim, Hyeon Jeong Kim, Min-Kyu Yun, Seunghun Lee, Dae-Jung Kang

**Affiliations:** 1 Medical Food Team, MNH Bio Co., Ltd., Hwaseong-si, Gyeonggi 18469, Republic of Korea; 2 Biohealthcare R&D Center, HYUNDAI BIOLAND Co., Ltd., Ansan, Gyeonggi 15407, Republic of Korea

**Keywords:** *Limosilactobacillus fermentum*, hangover, alcohol, acetaldehyde, acute hangover scale, alcohol hangover severity scale

## Abstract

Hangover refers to various mental and physical side effects following alcohol consumption. This study investigated the efficacy of paraprobiotic *Limosilactobacillus fermentum* HDB1098 (MB-LFH1098, Pharmavio ALC) on improving hangover symptoms and was designed as a randomized double-blind crossover placebo-controlled clinical trial conducted with 28 participants. Participants consumed 42.8% whiskey with equivalent to 90 g of alcohol and serum alcohol and acetaldehyde levels were measured at 0, 0.25, 0.5, 1, 2, 4, 6, and 15 h after alcohol consumption. Both serum alcohol (*p* < 0.01) and acetaldehyde (*p* < 0.05) levels over time, AUC, C_max_, and T_max_ for serum acetaldehyde level were significantly lower (*p* < 0.05) in the HDB1098 group compared to the placebo group. Furthermore, the total scores of AHS and AHSS were significantly lower (*p* < 0.0001) in the HDB1098 group than the placebo group, with improvement of several indicators in each questionnaire. These findings indicate that HDB1098 administration can effectively contribute to relief of hangover symptoms.

## Introduction

Hangover, uncomfortable physiological and psychological symptoms such as headache, tachycardia, stomachache, nausea, and neurocognitive impairment, follows heavy alcohol drinking and generally begins when blood alcohol concentration (BAC) is > 0.08% [1, 2]. Alcohol is metabolized in the liver by alcohol dehydrogenase (ADH) into the acetaldehyde, which is then rapidly oxidized into acetate by acetaldehyde dehydrogenase (ALDH) to be excreted as carbon dioxide (CO_2_) and urine [2]. However, when acetaldehyde is accumulated due to impaired alcohol metabolism, it serves as a primary contributor to various hangover symptoms leading to not merely individual health issues but also trigger for significant social and economic problems by reducing productivity [1, 3]. Consequently, the demand for effective methods to treat and prevent hangovers has been steadily increasing.

Some commercialized painkillers, including aspirin and ibuprofen, are usually utilized for symptomatic relief on alcohol consumption-induced headache and myalgia, and antacids are also able to be applicated to mitigate gastritis and nausea after drinking. However, these medications should be cautiously used because its hepatotoxicity can be provoked by alcohol metabolism during hangover [1]. Instead of these drugs, hangover cure products such as vitamin B1, B6, milk thistle extract, pear juice, and caffeine have been steadily popular in the market with easy accessibility to consumption [4, 5]. Nevertheless, some of the registered products in the market have not been scientifically established about its anti-hangover effect and safety [4]. For these reasons, well-defined and safe novel ingredients mitigating hangover symptoms must be developed. Although various research articles demonstrate anti-hangover effects of natural products including *Hovenia dulcis* extract, phytoextract mixture (*Mesembryanthemum crystallinum*, *Pueraria lobata* flower, and *Artemisia indica*), yeast extract containing glutathione, and *Phyllanthus amarus* extract [6-9], and probiotics such as *Bifidobacterium* and *Leuconostoc* in the clinical study [10, 11], those of paraprobiotics, novel derivatives of probiotics, have not been fully explored yet.

Paraprobiotics are elucidated as inactivated probiotics that provide health-beneficial effects to the host and widely applied on industry because of various industrial advantages such as more improved safety and stability than probiotics [12]. In the previous study, we evaluated alleviating hangover and hepatoprotective effects of heat-killed paraprobiotic *Limosilactobacillus fermentum* HDB1098 *in vitro* and *in vivo* [13]. HDB1098 significantly reduced acetaldehyde-induced hepatotoxicity in HepG2 cells by regulating pro-/anti-inflammatory cytokines (TNF-α, IL-1β, and IL-10) gene expressions, and also significantly diminished blood acetaldehyde concentration via significant stimulation of hepatic ALDH activity, as well as slight increase of ADH activity in alcohol-induced Sprague-Dawley rats [13]. Our previous results suggest that heat-killed paraprobiotic *L. fermentum* HDB1098 has the possibility for novel hangover improvement ingredients.

Based on the previous findings, this clinical study aimed to evaluate the efficacy of HDB1098 on hangover alleviation in healthy adults after alcohol consumption.

## Materials and Methods

### Ethics Statement

This study was conducted in accordance with the Declaration of Helsinki with approval from the Ethicare Ethics Committee of India (IRB No. IORG0009526, 9 July 2024), in Mumbai, India, and retrospectively registered with the Clinical Trials Registry – India (CTRI/2025/03/082959) on 15 March 2025.

### Subjects

This clinical trial was designed as a randomized controlled trial (RCT) and conducted from June to December 2024 in India. All methodologies were complied with the Guidelines for Human Clinical Trials of Hangover Cure Claims by the Ministry of Food and Drug Safety of Korea [14], and participants for this study were recruited from Raptim Research Pvt. Ltd. (India). Researchers provided all participants with detailed explanations of the study’s purpose and procedures, and written informed consent was obtained from all participants. The inclusion criteria were as follows: (1) healthy adults aged 18 to 45 years old, (2) a body mass index (BMI) between 18.5 to 29.9 kg/m^2^,(3) a history of experiencing hangovers after alcohol consumption, and (4) individuals who comprehended the study and voluntarily agreed to participate in the study. The exclusion criteria as follows: (1) individuals with a history of cardiovascular, immune, respiratory, gastrointestinal, diabetes, kidney, liver, biliary tract, urinary, nervous, or thyroid diseases, infections, malignant tumors, peptic ulcers, or gastrointestinal surgery, (2) pregnant or nursing women those planning to become pregnant, (3) individuals who have alcohol use disorders, alcoholism, or a positive result on a substance abuse qualitative test, (4) current user of anti-abuse drugs, herbal medicines, or hormonal contraceptives (for female participants), (5) those who have abnormal levels of creatinine, aspartate aminotransferase (AST), or alanine transaminase (ALT), and (6) individuals with reactive tests for human immunodeficiency virus (HIV) type I/II antibodies, hepatitis B surface antigen (HBsAg), or hepatitis C virus antibodies.

### Investigational Product

The investigational product (HDB1098) in this study was encapsulated in a 450 mg of opaque capsule containing 150 mg (33%) of medium-brown powder, which is composed of paraprobiotic *L. fermentum* HDB1098 (MB-LFH1098, Pharmavio ALC) and 300 mg (67%) of corn starch. Thus, participants consumed 1.0 × 10^12^ heat-killed *L. fermentum* cells/day which is a corresponding human equivalent dose based on the statistically significant effective dosage in the preclinical study. The placebo, indistinguishable in appearance and form to the investigational products, consisted of 450 mg (100%) of corn starch. The study followed a crossover design, involving a single intake of HDB1098 and the placebo with a 7-day washout period between treatments.

### Study Design

This clinical trial was designed as a randomized, double-blind, crossover, placebo-controlled trial with 1:1 allocation ratio. Thirty participants were selected following screening tests to determine their eligibility for inclusion in the study and randomly allocated to either Sequence I or Sequence II using the PROC PLAN procedure in SAS^®^ Version 9.4 (SAS Institute Inc., USA) by Raptim Research Pvt. Ltd., and each was assigned a unique subject identification number. Each participant served as their own control, receiving both treatments sequentially. Both participants and investigators remained blinded to the intervention assignments until the conclusion of the study, and the randomization table and associated lists were maintained independently by the randomization manager. A one-week washout period was implemented between Sequence I and Sequence II to eliminate any potential carry-over effects.

### Interventions and The Alcohol Challenge Test

All participants received a standardized meal prior to the trial. Water intake was restricted for the following 2 h, after which they were administered either placebo or HDB1098 capsules with 240 ml of water. Thirty minutes after capsule ingestion, participants consumed 42.8% of whiskey (equivalent to 90 g of alcohol) with the total volume divided into three equal portions (88.72 ml each). They were permitted to dilute the whiskey with water if desired and were instructed to consume each portion within 15 min, thereby completing the total ingestion within 45 min. Following consumption, participants stayed overnight at the clinical research center. Upon awakening the next morning, they completed a hangover symptom questionnaire at 12 h and 24 h after alcohol consumption. In addition, they also underwent physical examinations, vital sign assessments, and clinical laboratory tests.

### Assessment of Hangover Severity

A short-term intervention study was conducted to assess the severity of hangover symptoms using questionnaires. The efficacy of hangover relief was evaluated with the AHS (Alcohol Hangover Scale) and AHSS (Alcohol Hangover Severity Scale), both of which have been widely used in previous studies and are well-established, reliable assessment tools [15, 16]. The AHS includes nine items: ‘hangover’, ‘thirsty’, ‘tired’, ‘headache’, ‘dizziness/faintness’, ‘loss of appetite’, ‘stomachache’, ‘nausea’, and ‘heart racing’. Each item is scored on a scale from 0 to 7, with the following anchors: ‘none’ (0), ‘mild’ (1), ‘moderate’ (4), and ‘incapacitating’ (7). Data from AHS assessments were collected at 1 h and 12 h after alcohol consumption.

The AHSS comprises 12 items: ‘fatigue’, ‘apathy (lack of interest/concern)’, ‘concentration of problems’, ‘clumsiness’, ‘confusion’, ‘thirst’, ‘sweating’, shivering’, ‘stomach pain’, ‘nausea’, ‘dizziness’, and ‘heart pounding’. Each item is rated on a scale from 0 to 10, where 0 indicates the absence of symptoms and 10 represents extreme symptoms. Data from AHSS assessments were collected at 1 h and 24 h after alcohol consumption.

### Safety and Adverse Events

Vital signs, including blood pressure (systolic and diastolic), body temperature, and pulse rate, as well as clinical laboratory tests (hematology, biochemical, and urine analyses), were assessed on the participants. Adverse events were verified through participant interviews or questionnaires throughout the study.

### Serum Alcohol and Acetaldehyde Level Analysis

A total of 5 ml venous blood samples were collected from each participant at 0, 0.25, 0.5, 1, 2, 4, 6, and 15 h after alcohol consumption using venipuncture. To enable repeated sampling, a saline-locked angiocatheter was inserted into the vein, and 5 ml of blood was drawn at each time point. The collected samples were transferred into VACUETTE blood collection tubes (Greiner Bio-One, Austria) and stored at 4°C for 30 min. Subsequently, the samples were centrifuged at 3,000 rpm for 10 min at 4°C, and the separated sera were aliquoted and stored at –70°C until analysis.

Serum alcohol and acetaldehyde concentrations were quantified using gas chromatography–tandem mass spectrometry (GC–MS/MS; Agilent 8890/7000E, Agilent Technologies, USA) equipped with an autosampler (COMBI–PAL RSI 85). A DB-624plus column (60 m × 0.25 mm i.d., 1.4 μm film thickness; Agilent Technologies, USA) was employed. Acetonitrile was used as the internal standard, and calibration curves were established to ensure quantification accuracy.

Pharmacokinetic parameters, including C_max_, T_max_, and AUC, for alcohol and acetaldehyde were determined for each participant. C_max_ and T_max_ were defined as the highest observed serum concentration and the corresponding time point, respectively, without interpolation. AUC was calculated using the linear trapezoidal rule as implemented in GraphPad Prism 6.0 (GraphPad Software, USA). Statistical comparisons between the HDB1098 and placebo groups were conducted using the Wilcoxon signed-rank test, with *p* < 0.05 considered statistically significant.

### Statistical Analysis

Statistical analyses were conducted using the GraphPad Prism 6.0 (GraphPad Software Inc.) and data are presented as the means ± standard deviation (SD). Paired comparisons between treatments were conducted within each participant, consistent with the crossover design. Within-group comparisons were performed using a paired *t*-test, and when data were not normally distributed, the Wilcoxon signed-rank test was applied for within-subject comparisons. Over-time changes in serum levels within groups were analyzed using the two-away ANOVA. *P* < 0.05 is considered as statistically significant.

## Results

### Enrollment

**Fig. 1:**
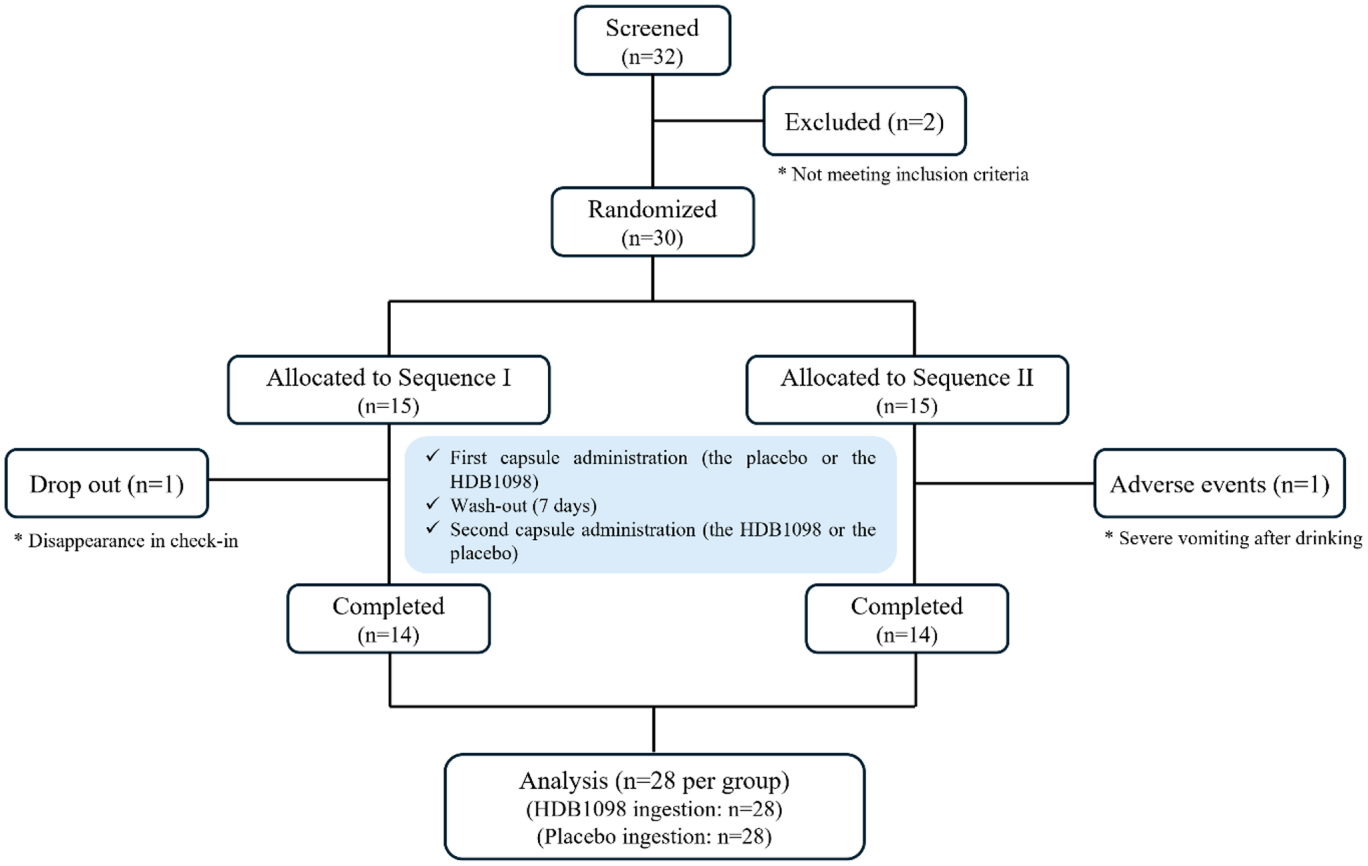
A flowchart for the allocation of participants in this study.

Thiry-two participants were initially enrolled; after excluding two during screening, 15 participants were allocated to each of the Sequence I and Sequence II. During the trial, two participants were excluded from the final analysis; one from Sequence I did not appear at the check-in, and one from Sequence II withdrew the trial due to severe vomiting after alcohol consumption (Fig. 1). Demographic and efficacy data analysis was based on the per-protocol (PP) set including 28 participants (14 from Sequence I and 14 from Sequence II) while safety analysis was conducted in safety set including 30 participants who took capsules at least once.

### Demographic Characteristics

The demographic information of the participants is shown in Table 1. Participants had a mean age of 31.39 ± 4.76 years, a mean weight of 63.99 ± 11.13 kg, and a body mass index (BMI) of 24.36 ± 3.77 kg/m^2^. These values may be considered slightly above the normal weight range according to World Health Organization standards [15], but vital signs, including systolic and diastolic blood pressure, pulse, and respiratory rate, were within normal limits. Participants reported an average alcohol consumption of 9.89 ± 3.44 drinks per week. According to the Centers for Disease Control and Prevention (CDC) [18], moderate alcohol use is defined as up to 2 drinks per day, or 14 drinks per week, indicating that the participants met the inclusion criteria for occasional or moderate alcohol consumption.

### Safety Evaluation

Safety evaluation was performed with safety set analysis at 36 h after alcohol consumption (Table 2). Statistically significant differences were observed in several hematological examination items. The white blood cell (WBC) analysis during the blood chemistry examination revealed the following results. The WBC concentration in the HDB1098 group was 6,606.07 ± 1,414.52 per cubic mm, which was significantly lower than the placebo group (7,197.50 ± 1,785.63 per cubic mm, *p* = 0.0223). The eosinophil concentration in the HDB1098 group was 37.90 ± 7.48%, significantly higher compared to the placebo group (3.99 ± 2.99%, *p* = 0.0082). The alkaline phosphatase (ALP) level in the HDB1098 group was 247.89 ± 51.42 U/L, which was also significantly higher than that of the placebo group (215.30 ± 50.14 U/L, *p* = 0.0050). However, this change was within the normal range [19], and no clinical significance was identified. Furthermore, no significant differences in adverse reactions were observed between the groups, and no severe adverse reactions occurred, thereby confirming that HDB1098 was well-tolerated with no adverse effects reported.

### Survey of Hangover Symptoms

The AHS (1 h and 12 h after alcohol consumption) and AHSS (1 h and 24 h after alcohol consumption) surveys were performed to evaluate relieving effect of HDB1098 against various hangover symptoms in participants. Total AHS scores in the HDB1098 group were 1.04 ± 1.14 at 1 h and 3.46 ± 1.79 at 12 h after alcohol intake with remarkably significant differences between the groups (*p* < 0.0001), while those in the placebo group were 3.07 ± 1.65 at 1 h and 7.68 ± 2.75 at 12 h after drinking. In comparison with the placebo group, 2 items (‘dizziness/faintness’, and ‘nausea’) were significantly improved at 1 h after alcohol intake, and 5 items (‘hangover’, ‘tired’, ‘dizziness/faintness’, ‘loss of appetite’ and ‘nausea’) showed significantly lower scores at 12 h after alcohol consumption in the HDB1098 group (Table 3).

Total AHSS scores at 1 h post-alcohol intake were 2.21 ± 1.10 in the HDB1098 group and 3.21 ± 2.30 in the placebo group, and those at 24 h post-alcohol intake were 3.21 ± 2.30 in the HDB1098 group and 7.79 ± 3.33 in the placebo group, showing a highly significant difference between the groups (*p* < 0.0001) similarly to the AHS survey. In the HDB1098 group, ‘nausea’ and ‘dizziness’ were significantly improved at 1 h after alcohol intake, and ‘fatigue’, ‘apathy’, ‘concentration problem’, ‘clumsiness’, ‘thirst’, ‘stomach pain’, ‘nausea’, and ‘dizziness’ were significantly relieved at 24 h after alcohol intake compared to the placebo group. These results demonstrate that HDB1098 is found to be effective in alleviating and improving hangover symptoms based on subjective assessments (Table 4).

### Alcohol and Acetaldehyde Levels

The peak alcohol serum concentration (C_max_), time to reach the C_max_ (T_max_), and area under the curve (AUC) for serum alcohol and acetaldehyde levels were shown in Table 5. For serum alcohol level, AUC, C_max_ and T_max_ in the HDB1098 group were reduced compared to those in the placebo group although the differences were not significant. For serum acetaldehyde level, C_max_ was significantly reduced in the HDB1098 group (189.18 ± 112.27 ppb) to those in the placebo group (272.92 ± 250.06 ppb) (*p* < 0.05) and T_max_ was also lower in the HDB1098 group (2.67 ± 2.2 h) than the placebo group (3.1 ± 2.0 h) with statistical significance (*p*<0.05). Furthermore, AUC for serum acetaldehyde level was 647.51 ± 374.43 ppb·h in the HDB1098 group and 820.42 ± 585.88 ppb·h in the placebo group. This was significantly lower in the HDB1098 group than in the placebo group (*p* < 0.05), suggesting that HDB1098 may mitigate the physiological effects of alcohol consumption and decrease hangover symptoms by modulating serum acetaldehyde levels.

**Fig. 2:**
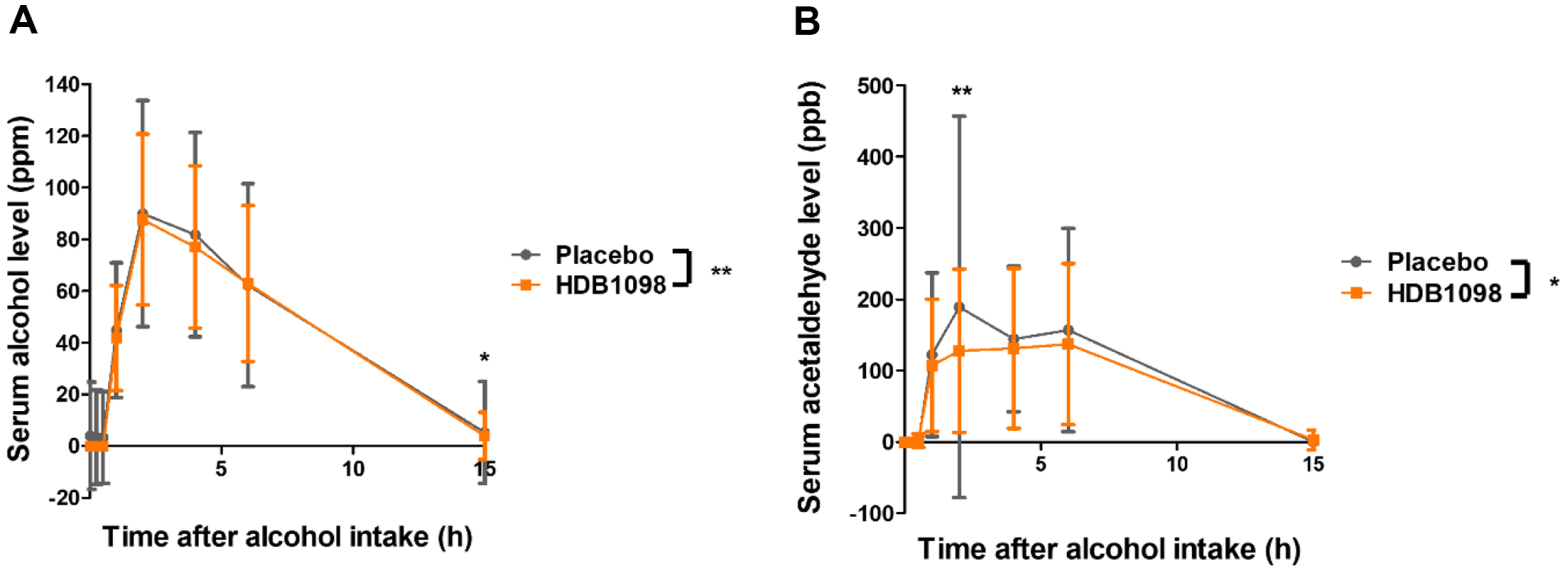
Serum alcohol and acetaldehyde levels. (**A**) Serum alcohol level (ppm) and (**B**) serum acetaldehyde level (ppb) after alcohol consumption. Data are presented as the mean ± SD. Compared between groups: *p*-value for the two-way ANOVA (*, *p* < 0.05; **, *p* < 0.01).

Serum alcohol level over time was significantly lower in the HDB1098 group than the placebo group (*p* < 0.01)(Fig. 2A). In particular, serum alcohol level at 15 h after alcohol consumption in the HDB1098 group (3.94 ± 9.11ppm) was significantly decreased compared to the placebo group (5.27 ± 19.68 ppm) (*p* <0.05). Serum acetaldehyde level over time was significantly reduced in the HDB1098 group compared to the placebo group (*p* < 0.05) with significant differences at 2 h after alcohol consumption; 127.73 ± 114.39 ppb in the HDB1098 group and 189.06 ± 267.44 ppb in the placebo group (*p* < 0.01) (Fig. 2B). All these results demonstrate that HDB1098 effectively reduced both serum alcohol and acetaldehyde levels with a greater decrease observed in acetaldehyde concentration.

## Discussion

This study aimed to provide clinical evidence of the effectiveness of paraprobiotic *L. fermentum* HDB1098 to improve hangover symptoms. HDB1098 was previously selected as a potent novel anti-hangover strain which exhibited hepatoprotective effect against acetaldehyde-induced hepatotoxicity and inflammation *in vitro* and decreased blood acetaldehyde concentration by enhancing hepatic ALDH activity *in vivo* [13]. The present study extends this evidence by suggesting the clinical efficacy of HDB1098 on improvement of hangover.

The AHS and AHSS surveys are valid questionnaires for subjective assessment of acute hangover symptoms after alcohol consumption in clinic [15, 16]. The total scores of AHS and AHSS showed significant differences between the HDB1098 group and placebo group, and several detailed categories were also markedly improved in the HDB1098 group. While few studies have reported significant differences in survey scores between probiotics and placebo group, and some studies-including those evaluating Duolac ProAP4 (a probiotics mixture consisting of *L. gasseri*, *L. casei*, *B. lactis*, and *B.breve*)- have even found no statistically significant effects [11], but some phytoextracts such as *P. amarus* have been reported to improve total scores and subitems in hangover symptom questionnaire after alcohol consumption similarly to the HDB1098 [7, 9]. This study is the first to clinically elucidate the beneficial effects of *L. fermentum* and its paraprobiotic form on the alleviation of hangover symptoms.

Ethanol (*i.e.*, alcohol) easily crosses the blood-brain barrier (BBB) and induces hangover symptoms via direct influence on central nervous system (CNS) [20]. Even though acetaldehyde cannot readily pass the BBB due to opulence of ALDH in BBB, it is also a key driver of hangover symptoms; acetaldehyde can produce toxic adducts by reacting with a lot of biomolecules such as DNA, proteins, neurotransmitters, and microtubules, resulting in tissue damage and functional impairment, etc. [21, 22]. Therefore, fast elimination of alcohol by facilitating alcohol metabolism plays a key role in reducing hangover severity [20]. In the present study, HDB1098 showed significant decreases in both serum alcohol and acetaldehyde levels compared to the placebo group over time, with more distinct differences in acetaldehyde levels. Pharmacokinetic markers (AUC, C_max_, and T_max_) also demonstrated that HDB1098 effectively facilitated clearance of blood alcohol and acetaldehyde, meaning the accelerating alcohol metabolism. Whether paraprobiotics administration decreases blood alcohol and acetaldehyde levels after drinking in clinic has not been fully established yet, but some research has suggested that supplementation of probiotics reduced concentration, AUC, and often C_max_ of blood acetaldehyde after alcohol consumption similarly to our findings [10, 11]. Interestingly, the present study aligned with our preclinical study; HDB1098 alleviated acetaldehyde-induced hepatic inflammation by down-regulating *TNFA* and *IL1B* and enhancing *IL10* expressions, which can cause loss of appetite and concentration problem after drinking [23], in HepG2 cells [13]. Furthermore, HDB1098-administered SD rats showed significantly lower blood acetaldehyde level than control group as similar to the observed reductions in the present study [13]. These preclinical and clinical results collectively suggest that HDB1098 effectively manages alcoholic stress and damage by facilitating acetaldehyde clearance as well as alcohol elimination after alcohol consumption.

The rate of alcohol metabolism is affected by activity of ADH and ALDH [22]. Jeong *et al*. have reported that supplementation of KISLipR, containing ALDH, significantly improved hangover symptoms in healthy men [24]. Jung *et al*. also have suggested that HY_IPA, a phytoextract mixture including *M. crystallinum*, *P. lobata* flower, and *A. indica*, enhanced ADH and ALDH activities thereby mitigating hangover in healthy adults [7]. Srinivasan *et al*. have demonstrated that *Pyrus* sp., including *Pyrus pyrifolia*, commonly used in South Korea for hangover relief, exhibited remarkable enzymatic activities (22.1% for ADH and 91.0% for ALDH) among various fruits and vegetables [25, 26]. These previous studies suggest the importance of ADH and ALDH activities in managing hangover symptoms. Although the present study did not directly assess the enzymatic activities of ADH and ALDH in participants, our preclinical research demonstrated that HDB1098 significantly promoted hepatic ALDH activity as well as moderate increase in hepatic ADH activity in alcohol-induces SD rats [13]. Based on these findings, it can be hypothesized that the observed effects in this study are attributable to the ability of HDB1098 to enhance both of ADH and ALDH activities, thereby accelerating the clearance of alcohol and acetaldehyde and alleviating hangover symptoms.

While the primary effects of HDB1098 appear to involve modulation of hepatic enzymes such as ADH and ALDH, recent evidence also suggests that probiotics may exert their beneficial effects via the gut-liver axis [27]. By improving gut microbiota composition and gut barrier function, HDB1098 may influence systemic inflammation and hepatic metabolic responses to alcohol. Therefore, the observed benefits in alcohol metabolism and acetaldehyde clearance could be attributed to both direct enzymatic regulation in the liver and indirect effects mediated through gut-liver axis modulation.

The safety profile of HDB1098 further supports its potential for clinical application. The WBC count in the HDB1098 group was significantly lower than that in the placebo group, whereas the levels of eosinophils and ALP were significantly higher. The observed decrease in WBC count may suggest a reduction in alcohol-induced systemic inflammatory response characterized by secretion of inflammatory cytokines [23, 28], and downregulatory effect of HDB1098 on alcoholic inflammation was supported by our preclinical study [13]. Reduction of eosinophils is accompanied by WBC increase due to inflammatory response, and Oltman *et al*. have reported that alcohol users have somewhat lower eosinophils count than normal persons [29, 30]. Thus, HDB1098-induced increase of eosinophils also indicates anti-inflammatory effect of HDB1098 against alcoholic inflammation. ALP is an enzyme associated with various metabolic activities in the liver, bones, and intestines, and increased ALP activity in the HDB1098 group can reflect the normalization of hepatic function because ALP activity tends to significantly decrease following alcohol consumption [31]. Therefore, all observed changes were spontaneously accompanied by the hangover relief process, and that was even within the normal physiological range, underscoring the safety profile or HDB1098.

In conclusion, the present study adduces clinical evidence for hangover relieving efficacy of the heat-killed paraprobiotic *L. fermentum* HDB1098. HDB1098 effectively improves hangover symptoms in healthy adults by diminishing blood alcohol and acetaldehyde via ADH and ALDH activities, thereby facilitating alcohol elimination. Although further research with larger cohorts and longer durations is needed to clarify the underlying mechanisms, it is encouraging that the present study is the first to address the clinical efficacy of paraprobiotics for hangover management. Hence, HDB1098 may serve as a potential ingredient in the development of health-beneficial products aimed at alleviating alcohol-induced hangover symptoms.

## Figures and Tables

**Table 1:** Demographic characteristics of the participants at baseline (PP set).

Variables	Total (*n* = 28)^a^
Age (Years)	31.39 ± 4.76
Male (n, %)	18 (64.29)
Female (n, %)	10 (35.71)
Height (cm)	161.94 ± 7.73
Weight (kg)	63.99 ± 11.13
BMI (kg/m^2^)	24.36 ± 3.77
Systolic blood pressure (mm/Hg)	118.07 ± 10.41
Diastolic blood pressure (mm/Hg)	73.29 ± 5.92
Pulse (per minute)	73.50 ± 9.33
Respiratory rate (per minute)	16.64 ± 1.34
Alcohol consumption (drinks/week)^b^	9.89 ± 3.44
Race	Asian Indian (100)

^a^Values are expressed as the mean ± SD or number (%).

^b^Units are expressed according to the Centers for Disease Control and Prevention standards.

**Table 2:** Variation in blood biochemical and histological testing following alcohol challenge test.

	HDB1098 (*n* = 28)	Placebo (*n* = 28)	*p*-value^a^
Hb (g/dL)	13.57 ± 1.88	13.45 ± 1.99	0.2168
WBC (per cubic mm)	6,606.07 ± 1,414.52	7,197.50 ± 1,785.63	0.0223*
Neutrophils (%)	49.78 ± 8.00	51.39 ± 7.92	0.2609
Lymphocytes (%)	37.90 ± 7.48	37.28 ± 7.09	0.6552
Eosinophils (%)	4.93 ± 2.93	3.99 ± 2.99	0.0082**
Basophils (%)	0.58 ± 0.34	0.54 ± 0.37	0.9864
Monocytes (%)	6.82 ± 2.23	6.81 ± 1.80	0.6522
Platelet (per cubic mm)	288,357.14 ± 69,291.77	284,214.29 ± 63,959.11	0.8081
Erythrocyte (%)	4.91 ± 0.63	4.90 ± 0.70	0.4041
Creatinine (mg/dL)	0.94 ± 0.11	0.86 ± 0.10	1.2869
AST(GOT) (U/L)	26.89 ± 12.54	29.72 ± 21.96	0.0876
ALT(GPT) (U/L)	28.28 ± 19.48	25.90 ± 18.90	0.0650
BUN (mg/dL)	7.31 ± 1.077	7.30 ± 1.79	0.4931
Bilirubin (mg/dL)	0.81 ± 0.45	0.84 ± 0.32	0.3244
ALP (U/L)	247.89 ± 51.42	215.30 ± 50.14	0.0050**

Values are presented as the mean ± SD. Abbreviation: Hb, hemoglobin; WBC, white blood cell; AST, aspartate aminotransferase; ALT, alanine aminotransferase; BUN, blood urea nitrogen; ALP, alkaline phosphatase. ^a^Comparison between groups: *p*-value for the paired *t*-test. *, *p* < 0.05; **, *p* < 0.01.

**Table 3:** Acute hangover scale.

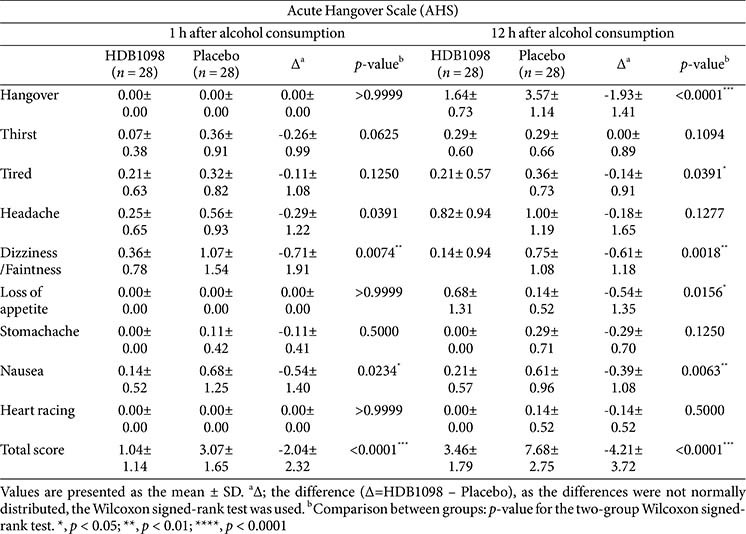

**Table 4:** Alcohol hangover severity scale.

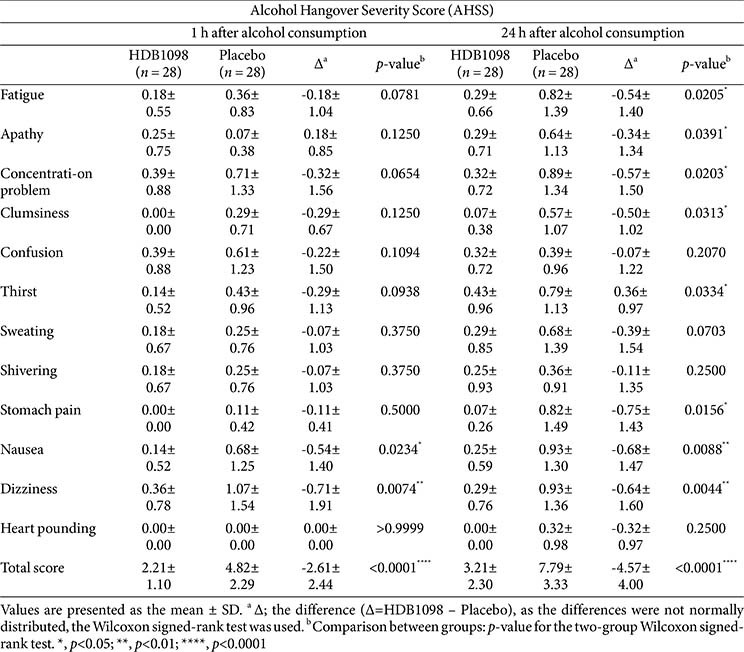

**Table 5:** Variation in serum alcohol and acetaldehyde level between HDB1098 and placebo groups.